# Supplementary material for: Profiling surface proteins on individual exosomes using a proximity barcoding assay
Source: Nat Commun. 2019 Aug 26;10:3854. doi: 10.1038/s41467-019-11486-1 (PMC6710248; doi:10.1038/s41467-019-11486-1)
Supplement: Supplementary file 1 — Supplementary Information [file 41467_2019_11486_MOESM1_ESM.pdf]

Supplementary information

Profiling surface proteins on individual exosomes using a proximity barcoding assay

Di Wu[1, 2, 3*], Junhong Yan[1], Xia Shen[4,5,6], Yu Sun[7], Måns Thulin[8] ,Yanling Cai[9], Lotta Wik[1], Qiujin Shen[1], Johan Oelrich[3], Xiaoyan Qian[2], K. Louise Dubois[10], K. Göran Ronquist[10], Mats Nilsson[2], Ulf Landegren[1], Masood Kamali-Moghaddam[1]

1. Department of Immunology, Genetics and Pathology, Science for Life Laboratory, Uppsala University, SE-75 185 Uppsala, Sweden.
2. Department of Biochemistry and Biophysics, Science for Life Laboratory, Stockholm University, SE-17 165, Solna, Sweden.
3. Vesicode AB, Uppsala, Sweden.
4. Center for Global Health Research, Usher Institute of Population Health Sciences and Informatics, University of Edinburgh, Edinburgh, UK
5. Department of Medical Epidemiology and Biostatistics, Karolinska Institutet, Stockholm, Sweden
6. Biostatistics Group, State Key Laboratory of Biocontrol, School of Life Sciences, Sun Yat-sen University, Guangzhou, China
7. Department of Molecular Evolution, Uppsala University, SE-751 24, Uppsala, Sweden.
8. Department of Statistics, Uppsala University, SE- 75 120 Uppsala, Sweden
9. Shenzhen Second People's Hospital, The First Affiliated Hospital of Shenzhen University, Shenzhen, China
10. Department of Medical Sciences, Clinical Chemistry, Uppsala University, SE-75 185 Uppsala, Sweden.

* Correspondence should be addressed to: di.wu@scilifelab.se

Keywords: prostasomes, exosomes, protein profiling, barcoding, rolling circle amplification, next generation sequencing

Supplementary note 1

Theoretical calculation and experimental analysis of erroneous identification of single complexes in PBA

In PBA, the risk to erroneously identify two or more individual complexes as a single complex lies in oligonucleotide design, assay procedure and data analysis. Here, we will address this problem by theoretical calculations and experimental investigations (the sample with complexes of STV-bio-oligonucleotides incubated separately at the molar ratio of 10:1 streptavidin to biotinylated oligonucleotides, with 32,525 identified complexes). If no false identification is present, all the identified complexes should carry a single proteinTag. In experimental data, we calculated the single complex percentage (Single %) as the number of complexes identified with one proteinTag among the total number of identified complexes to assess the level of false identification.

For oligonucleotide design, the number of nucleotides in complexTag (n) is crucial as it decides the diversity of the complexTags (4^n). As the probability of any given complexTag being used (k) by a total number of complexes (N) follows a Poisson distribution, $P\,(k\ events) = \lambda^{\kappa}\, e^{-\lambda}/k!$,or $F(x,\lambda)=\sum_{k=0}^{x} \lambda^k e^{-\lambda}/k!$, where $\lambda=N/4^n$. If we assume that the total number complexes is close to the experimentally obtained number (32,525), then we can calculate the percentage of complexTags used by one complex as the Single %=(F(1, λ)- F(0, λ))/ (1-F(0, λ)). We found that complexTags comprised of 12 random nucleotides would give a 99% of Single % in the calculation. In our experiment, we analyzed our results by using only the first 1 to 15 nucleotides as complexTag and the measured Single % fits closely the calculated values. (Supplementary figure 3).

Another circumstance leading to erroneous identification of separate complexes as single ones is when the density of complexes immobilized on the surface is too high so that some RCA products (RCP) might reach and hybridize to two or more complexes. For any circular area on the surface with the diameter of a RCP, the number of complexes located in this area follows a Poisson distribution $F(x,\lambda)=\sum_{k=0}^{x} \lambda^k e^{-\lambda}/k!$ where $\lambda = N/(S_{total}/S_{RCP})$. The total capturing surface $S_{total} \approx 25mm^2$. $S_{RCP}=0.25\times \pi D^2$, where D is the diameter of the RCP. By using different D values and numbers of complexes (N) we calculated the expected percentage of RCPs covering only one complex (Supplementary figure 4). The experimental data showed 99.27% of the 32,525 complexes carried only a single type of oligonucleotides when an excess of single species of oligonucleotides had been incubated separately with STV before combining complexes with four different oligonucleotides for analysis by PBA. From this we estimate that each RCP has a diameter of approximately 4 μm. This contribution to the risk of erroneous identification of separate complexes as single ones might be improved by decreasing RCP dimensions by shortening the time of RCA to generate the RCPs, optimizing the buffer composition to reduce the RCP size, or simply further diluting the RCPs and complexes.

It is also possible that some antibody and complex barcodes become artificially associated during PCR amplification. To reduce this risk, we incorporated dUTP during the RCA to generate the RCPs, and we then used UNG to cleave the RCPs before PCR to prevent them from serving as templates. Also, the use of T4 DNA polymerase with strong 3' exonuclease activity that degrade oligonucleotides on antibodies that have failed to be extended serves to prevent them from extending on other barcodes during PCR. However, it is still possible that a process of 'jumping' PCR may connect templates that were not linked before PCR. We expect that this risk mainly occurs towards the end of PCR, when PCR products reach higher concentrations. Therefore, the number of erroneously-joined barcoded products should be smaller than that of correctly barcoded products. The risk can be minimized by applying 'cutoff' values of sequencing reads to remove such low occurrence reads. We found that a cut-off of 2 amplicons sufficiently avoided this source of erroneous identification of members of two complexes as being part of one. Since a higher cut-off would require a greater sequencing depth, we applied a cutoff of 2 reads for all the analyses (Supplementary figure 5).

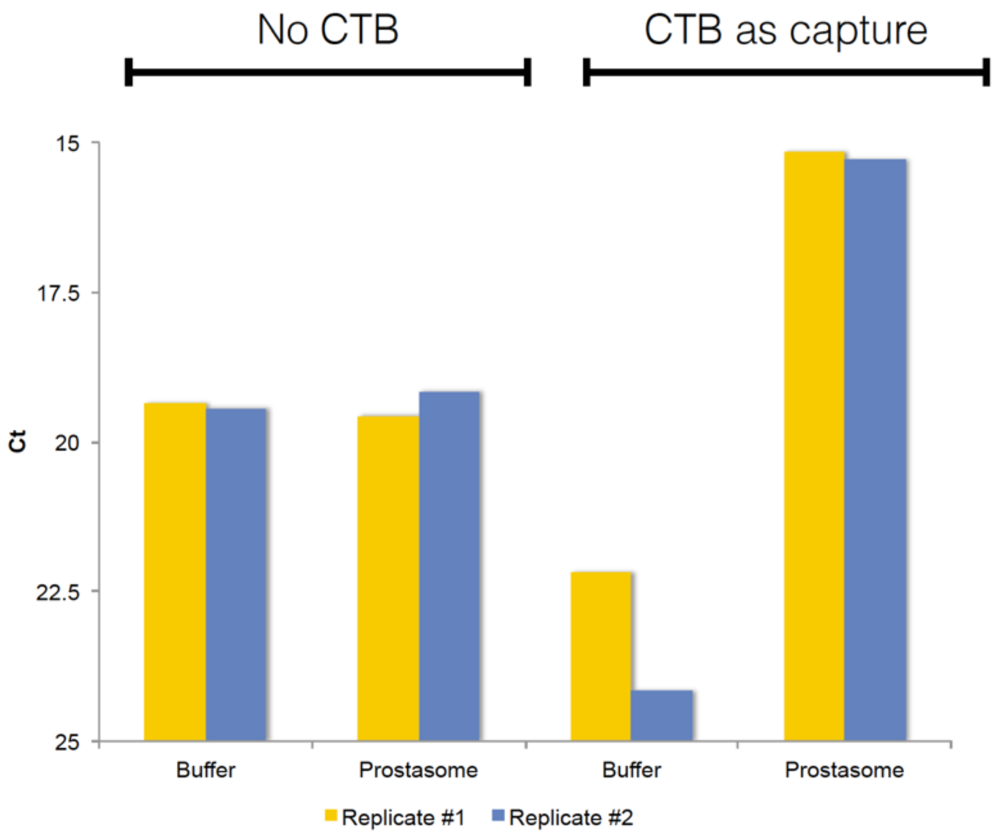

Supplementary Figure 1. Real-time PCR analysis of extension products of PBA in the presence or absence of CTB for capture. Prostasomes were incubated with PBA probes, then captured in STV-coated PCR tubes either with or without immobilized biotin-CTB. A buffer solution was also included as a negative control. After the extension of PBA probes on the RCA products, a pair of primers was used to amplify the extension products in real-time PCR. Cycle of threshold (Ct) values were plotted as bars. Duplicate measurements are shown as yellow or blue adjacent bars.

a

b

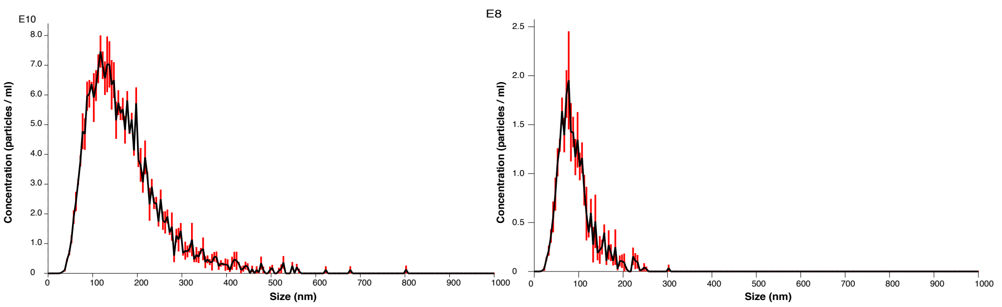

Supplementary Figure 2. Nanoparticle tracking analysis. (a) exosomes or (b) RCPs. were analyzed by nanoparticle tracking analysis. Averaged concentration vs sizes were plotted for each sample. Error bars indicate +/- standard deviation of the mean.

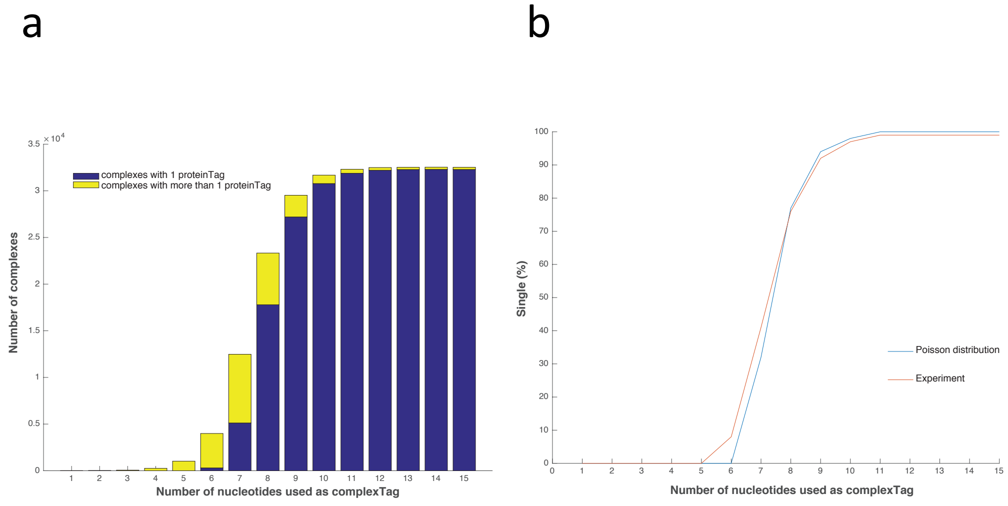

Supplementary Figure 3. Contribution to the risk of mistaking several clusters as one by failing to distinguish RCPs via their complexTags. (a) By analyzing from 1 to 15 nucleotides of the complexTags from the experimental data, the number of complexTags associated with one or more proteinTags was plotted against the number of nucleotides in the complexTag used for analysis. (b) The percentage of complexTags associated with a single proteinTag *versus* all complexes using experimental data from Figure 3a (red) is compared with theoretical calculation of the expected single percentage by using different length of complexTag (blue).

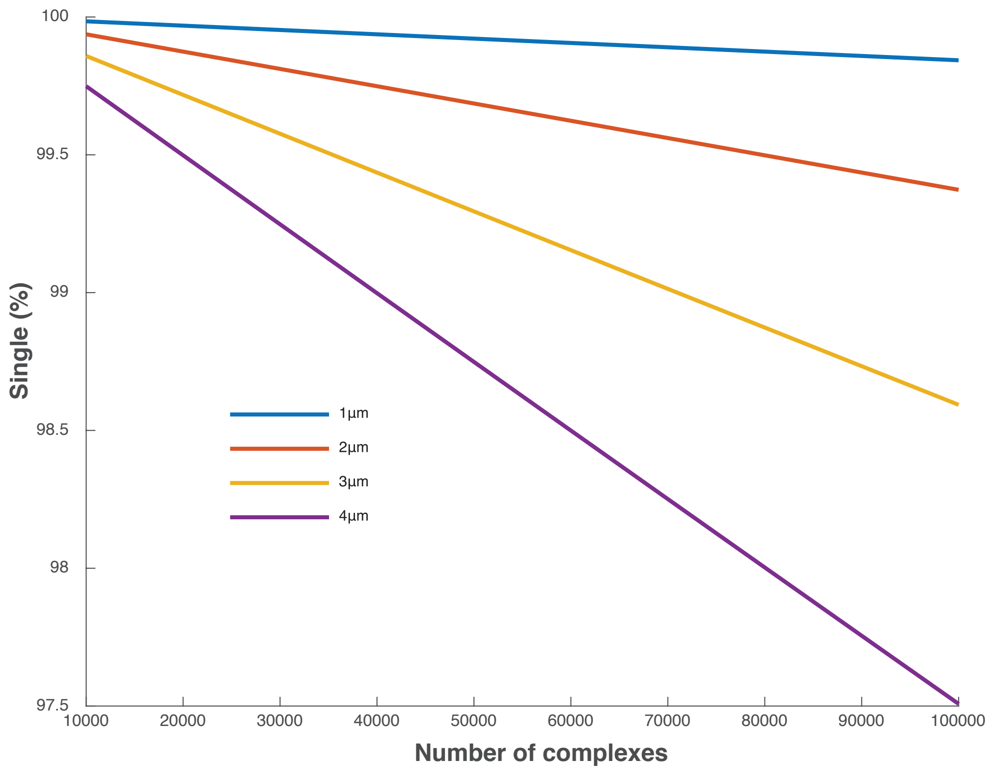

Supplementary Figure 4. The estimated risk of mistakenly identifying two or more complexes as one depending on the size of RCPs and the number of complexes. Using different sizes of RCPs and different numbers of complexes, theoretical percentage of RCPs covering only one complex was calculated using Poisson distribution.

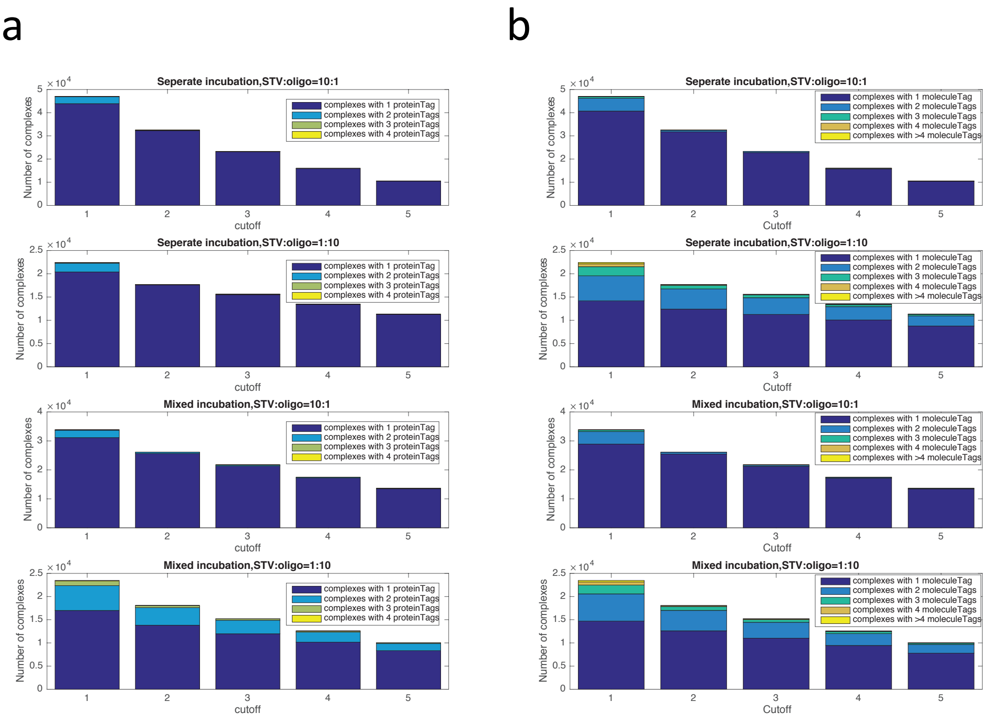

Supplementary Figure 5. Risk of mistakenly identifying two or more complexes as one during data analysis. Different cutoff values (numbers of reads for a given antibody-complex combination required for a positive identification) were applied to analyze the samples of STV-oligonucleotides experiments. The number of complexes with different (a) proteinTags or (b) moleculeTags are summarized.

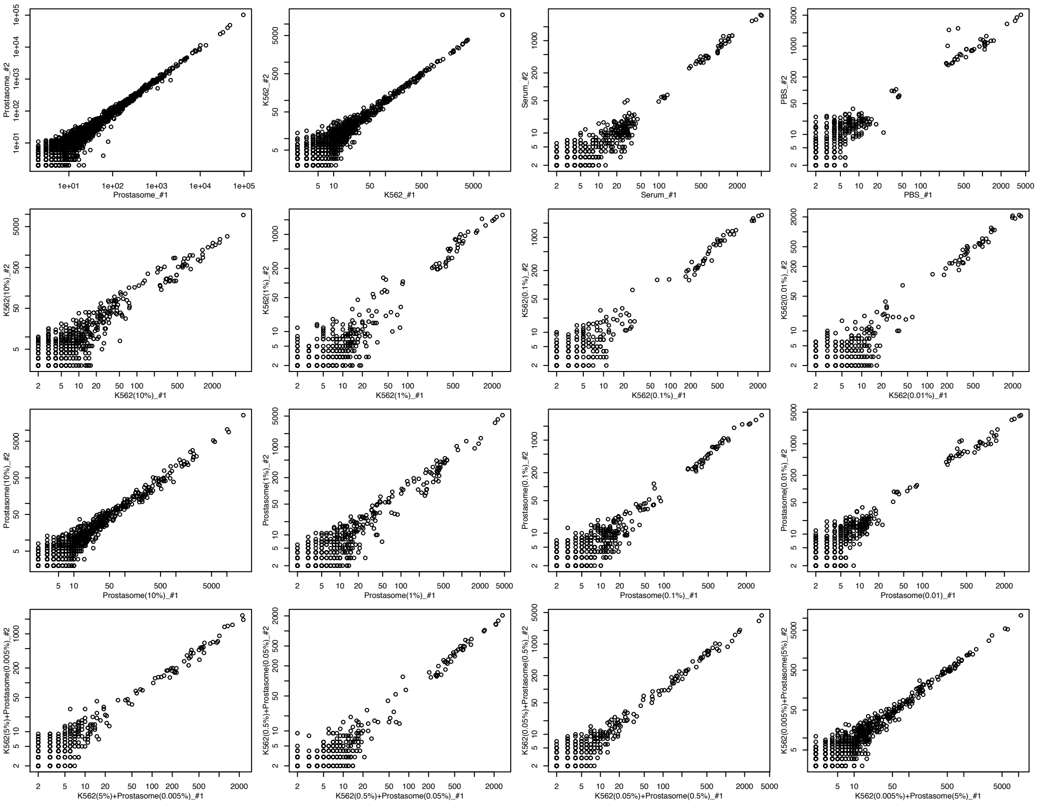

Supplementary Figure 6. Reproducibility of PBA for profiling exosome surface proteins. Numbers of exosomes identified with different combination of proteins in two independent runs are plotted as scatter plots.

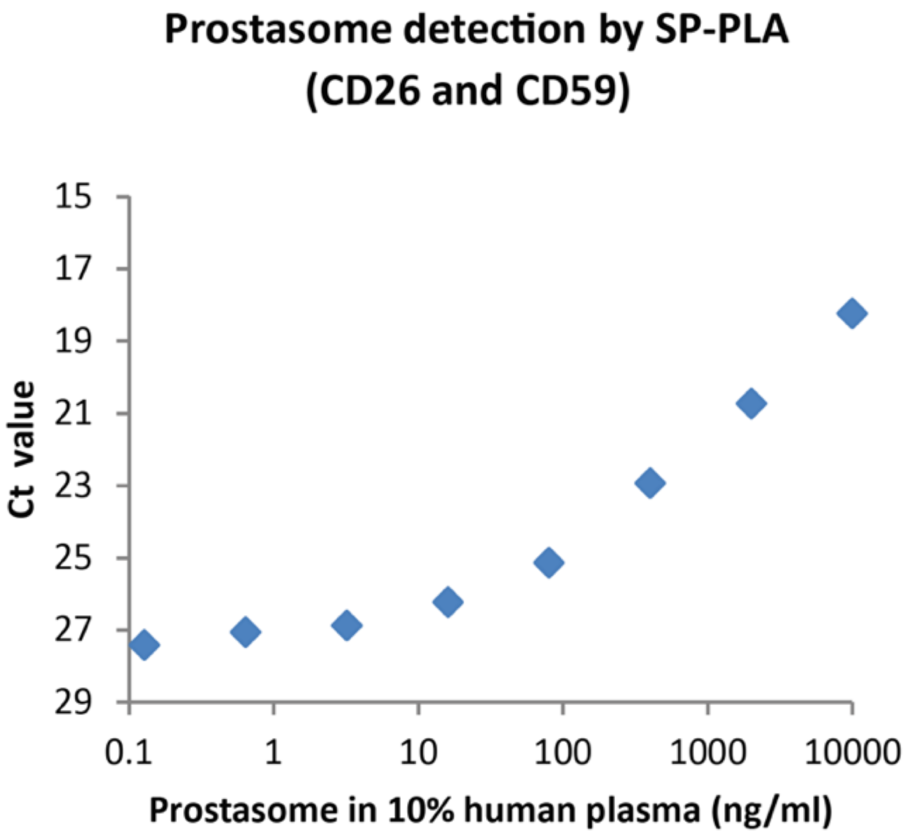

Supplementary Figure 7. Detection of a dilution series of prostasomes in 10% human plasma by SP-PLA, using CTB as capturing agent and a pair of PLA probes targeting CD26 and CD59

Supplementary Table 1. Oligonucleotides used in this study

Name	Sequence and modification
Ab-1	/5ThioMC6-D/AAGCGTGTGATTCCTAGTAATGAACAATGGNNNNNNNNTGCTATTATGATGTCTCAGGT
Ab-2	/5ThioMC6-D/AAGCGTGTGATTCCTAGTAATGAACCGAGTNNNNNNNNTGCTATTATGATGTCTCAGGT
Ab-3	/5ThioMC6-D/AAGCGTGTGATTCCTAGTAATGAACTGCATNNNNNNNNTGCTATTATGATGTCTCAGGT
Ab-4	/5ThioMC6-D/AAGCGTGTGATTCCTAGTAATGAAGATTCANNNNNNNNTGCTATTATGATGTCTCAGGT
Ab-5	/5ThioMC6-D/AAGCGTGTGATTCCTAGTAATGAAGGACTCNNNNNNNNTGCTATTATGATGTCTCAGGT
Ab-6	/5ThioMC6-D/AAGCGTGTGATTCCTAGTAATGAATATCGCNNNNNNNNTGCTATTATGATGTCTCAGGT
Ab-7	/5ThioMC6-D/AAGCGTGTGATTCCTAGTAATGACACCGTGNNNNNNNNTGCTATTATGATGTCTCAGGT
Ab-8	/5ThioMC6-D/AAGCGTGTGATTCCTAGTAATGACCAAGGTNNNNNNNNTGCTATTATGATGTCTCAGGT
Ab-9	/5ThioMC6-D/AAGCGTGTGATTCCTAGTAATGACCTGGTTNNNNNNNNTGCTATTATGATGTCTCAGGT
Ab-10	/5ThioMC6-D/AAGCGTGTGATTCCTAGTAATGACGCATGGNNNNNNNNTGCTATTATGATGTCTCAGGT
Ab-11	/5ThioMC6-D/AAGCGTGTGATTCCTAGTAATGACGGTCCTNNNNNNNNTGCTATTATGATGTCTCAGGT
Ab-12	/5ThioMC6-D/AAGCGTGTGATTCCTAGTAATGACTAGCGTNNNNNNNNTGCTATTATGATGTCTCAGGT
Ab-13	/5ThioMC6-D/AAGCGTGTGATTCCTAGTAATGAGATGGTCNNNNNNNNTGCTATTATGATGTCTCAGGT
Ab-14	/5ThioMC6-D/AAGCGTGTGATTCCTAGTAATGAGCCATGTNNNNNNNNTGCTATTATGATGTCTCAGGT
Ab-15	/5ThioMC6-D/AAGCGTGTGATTCCTAGTAATGAGCTCAATNNNNNNNNTGCTATTATGATGTCTCAGGT
Ab-16	/5ThioMC6-D/AAGCGTGTGATTCCTAGTAATGAGGTGACTNNNNNNNNTGCTATTATGATGTCTCAGGT
Ab-17	/5ThioMC6-D/AAGCGTGTGATTCCTAGTAATGATAGCAGCNNNNNNNNTGCTATTATGATGTCTCAGGT
Ab-18	/5ThioMC6-D/AAGCGTGTGATTCCTAGTAATGATCAGAGGNNNNNNNNTGCTATTATGATGTCTCAGGT
Ab-19	/5ThioMC6-D/AAGCGTGTGATTCCTAGTAATGATCGATATNNNNNNNNTGCTATTATGATGTCTCAGGT
Ab-20	/5ThioMC6-D/AAGCGTGTGATTCCTAGTAATGATCTGCGCNNNNNNNNTGCTATTATGATGTCTCAGGT
Ab-21	/5ThioMC6-D/AAGCGTGTGATTCCTAGTAATGATTACGTGNNNNNNNNTGCTATTATGATGTCTCAGGT
Ab-22	/5ThioMC6-D/AAGCGTGTGATTCCTAGTAATGCAAGGTAANNNNNNNNTGCTATTATGATGTCTCAGGT
Ab-23	/5ThioMC6-D/AAGCGTGTGATTCCTAGTAATGCAATGAAGNNNNNNNNTGCTATTATGATGTCTCAGGT
Ab-24	/5ThioMC6-D/AAGCGTGTGATTCCTAGTAATGCACAGTCTNNNNNNNNTGCTATTATGATGTCTCAGGT
Ab-25	/5ThioMC6-D/AAGCGTGTGATTCCTAGTAATGCACGTTCGNNNNNNNNTGCTATTATGATGTCTCAGGT
Ab-26	/5ThioMC6-D/AAGCGTGTGATTCCTAGTAATGCTAAGGTTNNNNNNNNTGCTATTATGATGTCTCAGGT
Ab-27	/5ThioMC6-D/AAGCGTGTGATTCCTAGTAATGCTGATTCGNNNNNNNNTGCTATTATGATGTCTCAGGT
Ab-28	/5ThioMC6-D/AAGCGTGTGATTCCTAGTAATGCTTAAGGANNNNNNNNTGCTATTATGATGTCTCAGGT
Ab-29	/5ThioMC6-D/AAGCGTGTGATTCCTAGTAATGGAATGTACNNNNNNNNTGCTATTATGATGTCTCAGGT
Ab-30	/5ThioMC6-D/AAGCGTGTGATTCCTAGTAATGGAGGTTCCNNNNNNNNTGCTATTATGATGTCTCAGGT
Ab-31	/5ThioMC6-D/AAGCGTGTGATTCCTAGTAATGGATGGACANNNNNNNNTGCTATTATGATGTCTCAGGT
Ab-32	/5ThioMC6-D/AAGCGTGTGATTCCTAGTAATGGGACAAGTNNNNNNNNTGCTATTATGATGTCTCAGGT
Ab-33	/5ThioMC6-D/AAGCGTGTGATTCCTAGTAATGTAATCGATNNNNNNNNTGCTATTATGATGTCTCAGGT
Ab-34	/5ThioMC6-D/AAGCGTGTGATTCCTAGTAATGTACTTGCGNNNNNNNNTGCTATTATGATGTCTCAGGT
Ab-35	/5ThioMC6-D/AAGCGTGTGATTCCTAGTAATGTCGACACCNNNNNNNNTGCTATTATGATGTCTCAGGT
Ab-36	/5ThioMC6-D/AAGCGTGTGATTCCTAGTAATGTGACCATGNNNNNNNNTGCTATTATGATGTCTCAGGT
Ab-37	/5ThioMC6-D/AAGCGTGTGATTCCTAGTAATGTGTCAGGANNNNNNNNTGCTATTATGATGTCTCAGGT
Ab-38	/5ThioMC6-D/AAGCGTGTGATTCCTAGTAATGTTACTCCGNNNNNNNNTGCTATTATGATGTCTCAGGT
Ab-39	/5ThioMC6-D/AAGCGTGTGATTCCTAGTAATGTGTCGACTNNNNNNNNTGCTATTATGATGTCTCAGGT
Ab-40	/5ThioMC6-D/AAGCGTGTGATTCCTAGTAATGTTAGACCTNNNNNNNNTGCTATTATGATGTCTCAGGT
P5-D501	AATGATACGGCGACCACCGAGATCTACACTATAGCCTACACTCTTTCCCTACACGAC
P5-D502	AATGATACGGCGACCACCGAGATCTACACATAGAGGCACACTCTTTCCCTACACGAC
P5-D503	AATGATACGGCGACCACCGAGATCTACACCCTATCCTACACTCTTTCCCTACACGAC
P5-D504	AATGATACGGCGACCACCGAGATCTACACGGCTCTGAACACTCTTTCCCTACACGAC
P5-D505	AATGATACGGCGACCACCGAGATCTACACAGGCGAAGACACTCTTTCCCTACACGAC
P5-D506	AATGATACGGCGACCACCGAGATCTACACTAATCTTAACACTCTTTCCCTACACGAC
P5-D507	AATGATACGGCGACCACCGAGATCTACACCAGGACGTACACTCTTTCCCTACACGAC
P5-D508	AATGATACGGCGACCACCGAGATCTACACGTACTGACACACTCTTTCCCTACACGAC
P7-D701	CAAGCAGAAGACGGCATACGAGATCGAGTAATGTGACTGGAGTTCAGACGTGT
P7-D702	CAAGCAGAAGACGGCATACGAGATTCTCCGGAGTGACTGGAGTTCAGACGTGT
P7-D703	CAAGCAGAAGACGGCATACGAGATAATGAGCGGTGACTGGAGTTCAGACGTGT
P7-D704	CAAGCAGAAGACGGCATACGAGATGGAATCTCGTGACTGGAGTTCAGACGTGT
P7-D705	CAAGCAGAAGACGGCATACGAGATTTCTGAATGTGACTGGAGTTCAGACGTGT
P7-D706	CAAGCAGAAGACGGCATACGAGATACGAATTCGTGACTGGAGTTCAGACGTGT

P7-D707	CAAGCAGAAGACGGCATACGAGATAGCTTCAGGTGACTGGAGTTCAGACGTGT
P7-D708	CAAGCAGAAGACGGCATACGAGATGCGCATTAGTGACTGGAGTTCAGACGTGT
P7-D709	CAAGCAGAAGACGGCATACGAGATCATAGCCGGTGACTGGAGTTCAGACGTGT
P7-D710	CAAGCAGAAGACGGCATACGAGATTTCGCGGAGTGACTGGAGTTCAGACGTGT
P7-D711	CAAGCAGAAGACGGCATACGAGATGCGCGAGAGTGACTGGAGTTCAGACGTGT
P7-D712	CAAGCAGAAGACGGCATACGAGATCTATCGCTGTGACTGGAGTTCAGACGTGT
Universal primer1	ACACTCTTTCCCTACACGAC
Universal primer2	GTGACTGGAGTTCAGACGTGTGCTCTTCCGATCTGCGTGTGATTCCTAGTAATG
P5	AATGATACGGCGACCACCGAGATCTACAC
P7	CAAGCAGAAGACGGCATACGAGAT
Padlock	5’/Phos/GCTATTATGATGTCTCAGGUNNNNNNNNNNNNNNNAGAUCGGAAGAGCGTCGTGTAGGGAAAGAGTGTCTAGTGCTGGATGATCGTCC
template	CTGAGACATCATAATAGCGGACGATCATCCAGCACT
Blocking oligo	CTAGTGCTGGATGATCGTCCmUmUmUmUmU(2’O-Methyl RNA base)
SLC1	5'/streptavidin/ttttttCATCGCCCTTGGACTACGACTAAATCGTG
SLC2	5'/phosphate/TCGTGTCTAAAGTCCGTTACCTTGATTtctctc/streptavidin/3’
BioFwd	CATCGCCCTTGGACTACGA
BioRev	GGGAATCAAGGTAACGGACTTTAG
BioSplint	TACTTAGACACGACACGATTTAGTTT
STV1*	5'/bio/AAGCGAAACCTGGTCCGGTATCTTGGACNNNNNNNNNNTGCTATTATGATGTCTCAGGT
STV2*	5'/bio/AAGCGAAACCTGGTCCGGTATCCTGTGTNNNNNNNNNNTGCTATTATGATGTCTCAGGT
STV3*	5'/bio/AAGCGAAACCTGGTCCGGTATCGGACATNNNNNNNNNNTGCTATTATGATGTCTCAGGT
STV4*	5'/bio/AAGCGAAACCTGGTCCGGTATCCAAAGTNNNNNNNNNNTGCTATTATGATGTCTCAGGT
P5-index1*	AATGATACGGCGACCACCGAGATCTACACTCTTTCCCTACACGACGCTCTTCCGATCTGGGTTTGCGAAACCTGGTCCGGTATC
P5-index2*	AATGATACGGCGACCACCGAGATCTACACTCTTTCCCTACACGACGCTCTTCCGATCTATGGCGGCGAAACCTGGTCCGGTATC
P5-index3*	AATGATACGGCGACCACCGAGATCTACACTCTTTCCCTACACGACGCTCTTCCGATCTTTCATAGCGAAACCTGGTCCGGTATC
P5-index4*	AATGATACGGCGACCACCGAGATCTACACTCTTTCCCTACACGACGCTCTTCCGATCTAACGCCGCGAAACCTGGTCCGGTATC
P5-index5*	AATGATACGGCGACCACCGAGATCTACACTCTTTCCCTACACGACGCTCTTCCGATCTGGCTGCGCGAAACCTGGTCCGGTATC
P5-index6*	AATGATACGGCGACCACCGAGATCTACACTCTTTCCCTACACGACGCTCTTCCGATCTGCTGTGGCGAAACCTGGTCCGGTATC
P5-index7*	AATGATACGGCGACCACCGAGATCTACACTCTTTCCCTACACGACGCTCTTCCGATCTAGATGGGCGAAACCTGGTCCGGTATC
P5-index8*	AATGATACGGCGACCACCGAGATCTACACTCTTTCCCTACACGACGCTCTTCCGATCTGTAATGGCGAAACCTGGTCCGGTATC
P7-index1*	CAAGCAGAAGACGGCATACGAGATCGGTCTCGGCATTCCTGCTGAACCGCTCTTCCGATCTGTATACGTTGTATAGTATTCGCGCAG
P7-index2*	CAAGCAGAAGACGGCATACGAGATCGGTCTCGGCATTCCTGCTGAACCGCTCTTCCGATCTTGTCACGTTGTATAGTATTCGCGCAG
P7-index3*	CAAGCAGAAGACGGCATACGAGATCGGTCTCGGCATTCCTGCTGAACCGCTCTTCCGATCTTGCGGAGTTGTATAGTATTCGCGCAG
P7-index4*	CAAGCAGAAGACGGCATACGAGATCGGTCTCGGCATTCCTGCTGAACCGCTCTTCCGATCTACGAGCGTTGTATAGTATTCGCGCAG
Universal primer1*	GCGAAACCTGGTCCGGTATC
Universal primer2*	GTTGTATAGTATTCGCGCAG
Padlock*	5'/phosphate/GCTATTATGATGTCTCAGGTAGATGGNNNNNNNNNNNNNNNUUCTGCGCGAATACTATACAACCTAGTGCTGGATGATCGTCC

*The sequences with * are used only for the streptavidin experiment.*
The sampleTag and proteinTag were underlined

Supplementary Table 2. Antibodies used in this study

Antibody target	Supplier	Catalog number
Del1	Biotechne	AF6046
CD151	Biotechne	MAB1884
ITGA1	Biotechne	AF5676
ITGA8	Biotechne	MAB6194
ITGAX	Biotechne	MAB1777
ITGB5	Biotechne	AF8035
CD147	Biotechne	MAB3195
ITGA3	Biotechne	MAB1345
ITGA11	Biotechne	MAB4235
ITGA2B	Biotechne	MAB7616
ITGB6	Biotechne	MAB4155
CD227	Biotechne	AF6298
ITGA5	Biotechne	MAB1864
ITGAE	Biotechne	AF1990
ITGB2	Biotechne	AF1730
ADAM10	Biotechne	MAB1427
ITGA6	Biotechne	MAB13501
ITGAL	Biotechne	MAB35951
CD318	Biotechne	AF2666
ITGB8	Biotechne	MAB4775
ITGB3	Biotechne	AF2266
ITGA2	Biotechne	MAB12332
ITGA7	Biotechne	MAB3518
ITGAV	Biotechne	MAB2528
ITGB4	Biotechne	MAB4060
CD166	Biotechne	AF1172
EGFR	Biotechne	AF231
ITGB1	Biotechne	MAB1778
CD90	Biotechne	AF2067
EpCAM	Biotechne	AF960
ITGAM	Biotechne	MAB1124
ITGA9	Biotechne	AF3827
CD107a	Biotechne	AF4800
CD13	Biotechne	AF2335
CD59	Biotechne	AF1987
CD26	Biotechne	MAB1180
CD9	BD bioscience	M-L13
CD63	BD bioscience	H5C6
