# Supplementary material for: Profiling surface proteins on individual exosomes using a proximity barcoding assay
Source: Nat Commun. 2019 Aug 26;10:3854. doi: 10.1038/s41467-019-11486-1 (PMC6710248; doi:10.1038/s41467-019-11486-1)
Supplement: Supplementary file 3 — Reporting Summary [file 41467_2019_11486_MOESM3_ESM.pdf]

nature research

Corresponding author(s): Di Wu

Last updated by author(s): 2019-Jun-22

Reporting Summary

Nature Research wishes to improve the reproducibility of the work that we publish. This form provides structure for consistency and transparency in reporting. For further information on Nature Research policies, see Authors & Referees and the Editorial Policy Checklist .

Statistics

For all statistical analyses, confirm that the following items are present in the figure legend, table legend, main text, or Methods section.

n/a	Confirmed	
x		The exact sample size (*n*) for each experimental group/condition, given as a discrete number and unit of measurement
	x	A statement on whether measurements were taken from distinct samples or whether the same sample was measured repeatedly
x		The statistical test(s) used AND whether they are one- or two-sided<br>*Only common tests should be described solely by name; describe more complex techniques in the Methods section.*
x		A description of all covariates tested
x		A description of any assumptions or corrections, such as tests of normality and adjustment for multiple comparisons
x		A full description of the statistical parameters including central tendency (e.g. means) or other basic estimates (e.g. regression coefficient) AND variation (e.g. standard deviation) or associated estimates of uncertainty (e.g. confidence intervals)
x		For null hypothesis testing, the test statistic (e.g. *F*, *t*, *r*) with confidence intervals, effect sizes, degrees of freedom and *P* value noted<br>*Give P values as exact values whenever suitable.*
x		For Bayesian analysis, information on the choice of priors and Markov chain Monte Carlo settings
x		For hierarchical and complex designs, identification of the appropriate level for tests and full reporting of outcomes
x		Estimates of effect sizes (e.g. Cohen's *d*, Pearson's *r*), indicating how they were calculated

Our web collection on statistics for biologists contains articles on many of the points above.

Software and code

Policy information about availability of computer code

Data collection	The CT values from qPCR were obtained by MxPro(Agilent).<br>The size distribution of particles were obtained by NanoSight NTA (Malvern Panalytical).
Data analysis	The BCL files for each sample were converted to fastq formats by using bcl2fastq (Illumina) with pair indexes. The t-SNE algorithm applied was implemented in the R package Rtsne, and all the parameters were set to default in our pipeline. Other in-house developed perl and R scripts used in are available upon request.

For manuscripts utilizing custom algorithms or software that are central to the research but not yet described in published literature, software must be made available to editors/reviewers. We strongly encourage code deposition in a community repository (e.g. GitHub). See the Nature Research guidelines for submitting code & software for further information.

Data

Policy information about availability of data

All manuscripts must include a data availability statement. This statement should provide the following information, where applicable:

- Accession codes, unique identifiers, or web links for publicly available datasets
- A list of figures that have associated raw data
- A description of any restrictions on data availability

The data supporting this study are available in Figshare. FASTQ files used in this study are available by DOI: 10.6084/m9.figshare.7956023. The protein abundance on individual exosomes of each sample are available by DOI: 10.6084/m9.figshare.7963742.

Field-specific reporting

Please select the one below that is the best fit for your research. If you are not sure, read the appropriate sections before making your selection.

☒ Life sciences ☐ Behavioural & social sciences ☐ Ecological, evolutionary & environmental sciences

For a reference copy of the document with all sections, see nature.com/documents/nr-reporting-summary-flat.pdf

Life sciences study design

All studies must disclose on these points even when the disclosure is negative.

Sample size	We performed duplicate measurement for the 'spike-in' experiments, where different amount of K562 or prostasomes in serum exosomes at different ratios.
Data exclusions	We excluded the sequence with only one read, which we hypothesized might come from in-PCR barcoding. More details are explained in the supplementary note and Figure S5.
Replication	The purpose of this study is a proof of concept of the assay rather than discovery. So the the experimental findings are not replicated.
Randomization	This wasn't relevant to our study.
Blinding	We didn't involve collect data from different groups, so blinding wasn't relevant to our study.

Reporting for specific materials, systems and methods

We require information from authors about some types of materials, experimental systems and methods used in many studies. Here, indicate whether each material, system or method listed is relevant to your study. If you are not sure if a list item applies to your research, read the appropriate section before selecting a response.

Materials & experimental systems

n/a	Involved in the study	
☐	☒	Antibodies
☐	☒	Eukaryotic cell lines
☒	☐	Palaeontology
☒	☐	Animals and other organisms
☒	☐	Human research participants
☒	☐	Clinical data

Methods

n/a	Involved in the study	
☒	☐	ChIP-seq
☒	☐	Flow cytometry
☒	☐	MRI-based neuroimaging

Antibodies

Antibodies used	The antibodies used in this study are listed inTable S2.
Validation	The vendor have the validation data for each antibody.

Eukaryotic cell lines

Policy information about cell lines

Cell line source(s)	KATOIII (ATCC® HTB-103), AGS (ATCC® CRL-1739), MMK7(RIKEN). The exosomes from other cell lines are from the vendor. We gave the catalog number for each.
Authentication	None of the celllines used are authorized.
Mycoplasma contamination	The cell lines KATOIII (ATCC® HTB-103), AGS (ATCC® CRL-1739), MMK7(RIKEN) were tested negative in mycoplasma contamination.
Commonly misidentified lines (See ICLAC register)	Not involved.
